# In Vivo ^31^P‐MRS of the Human Orthotopic Kidney at 3 Tesla

**DOI:** 10.1002/nbm.70406

**Published:** 2026-09-20

**Authors:** Eva Carlsson, Jan Weis, Maria K. Svensson, Maysam Jafar, Per Liss, Amir Sedigh

**Affiliations:** ^1^ Department of Medical Sciences, Renal Sciences Uppsala University Uppsala Sweden; ^2^ Department of Medical Physics Uppsala University Hospital Uppsala Sweden; ^3^ Uppsala Clinical Research Center Uppsala Sweden; ^4^ Philips Nordic Solna Stockholm Sweden; ^5^ Section of Radiology, Department of Surgical Sciences Uppsala University Hospital Uppsala Sweden; ^6^ Deptartment of Surgical Sciences, Transplantation Surgery Uppsala University Uppsala Sweden

**Keywords:** 3T, feasibility, healthy kidney, ISIS, phosphorus MRS

## Abstract

Phosphorus magnetic resonance spectroscopy (^31^P‐MRS) is a noninvasive in vivo tool for identification and quantification of phosphorus‐containing metabolites involved in cell membrane synthesis and degradation, as well as in the energy‐producing pathways essential for cell functions. However, the considerable depth of the orthotopic kidneys in the human body, combined with the inherently low sensitivity of ^31^P‐MRS, makes measurement of the spectra particularly challenging. Despite these difficulties, the acquisition of phosphorus spectra from normal orthotopic kidney is important for studies of kidney physiology and disease, as well as for evaluation of human allograft viability and function before and after renal transplantation. The aim of the present study was to investigate whether ^31^P spectra of orthotopic kidneys can be acquired with improved spectral quality within a clinically acceptable acquisition time using a contemporary 3T MR scanner. Spectra from healthy volunteers were obtained using a single‐voxel image‐selected in vivo spectroscopy (ISIS) sequence, combined with proton (^1^H) decoupling and nuclear Overhauser effect enhancement. We demonstrate that ^31^P spectra with improved spectral resolution and signal‐to‐noise ratio can be acquired on a clinical 3T scanner within an acceptable acquisition time. However, careful selection of the volume of interest is essential, taking into account the transmitter excitation profile and the chemical shift displacement of the voxels corresponding to different spectral components. The reference phosphorus spectra of healthy orthotopic kidney may serve as a valuable baseline for assessing alterations and changes in renal metabolism associated with disease and transplantation.

## Graphical abstract

^31^P‐MRS of healthy human orthotopic kidneys at 3T yielded improved spectral quality within a clinically feasible scan time. The resulting summed spectrum characterizes normal renal phosphorus metabolism and may serve as a reference for detecting metabolic alterations associated with kidney disease and transplantation.

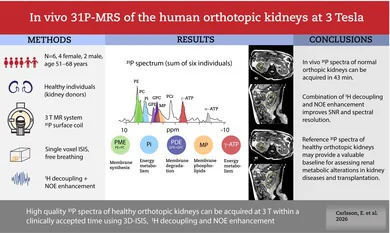

**Abbreviations**
AMARES: advanced method for accurate, robust and efficient spectral fitting
ATP: adenosine triphosphate
BTFE: balanced turbo field echo
CRLB: Cramér–Rao lower bound
CSDE: chemical shift displacement error
DPG: diphosphoglycerate
FID: free induction decay
GPC: glycerolphosphocholine
GPE: glycerolphosphoethanolamine
ISIS: image‐selected in vivo spectroscopy
jMRUI: Java‐based magnetic resonance user interface
MP: membrane phospholipid
NAD: nicotinamide adenine dinucleotide
NOE: nuclear Overhauser effect
PC: phosphocholine
PCr: phosphocreatine
PDE: phosphodiesters
PE: phosphoethanolamine
Pi: inorganic phosphate
PME: phosphomonoesters
SNR: signal‐to‐noise ratio

## 1 Introduction

Phosphorus magnetic resonance spectroscopy (^31^P‐MRS) is a noninvasive tool for the in vivo detection and quantification of phosphorus‐containing metabolites involved in cell membrane synthesis and degradation, as well as in energy‐producing pathways essential for cellular functions [1, 2, 3, 4]. The dominant signals in ^31^P spectra arise from intracellular metabolites, including phosphomonoesters (PME), inorganic phosphate (Pi), phosphodiesters (PDE), phosphocreatine (PCr), membrane phospholipids (MP), and the three phosphate groups (γ, α, and β) of adenosine triphosphate (ATP). Phospholipid metabolism is primarily reflected by the PME and PDE resonances. The PME intensity mainly comprises phosphocholine (PC) and phosphoethanolamine (PE), which serve as precursors of phosphatidylcholine and phosphatidylethanolamine, key components of cellular membrane phospholipids. Elevated PME concentration is generally associated with increased phospholipid metabolism, reflecting enhanced cell growth and/or proliferation. The PDE spectral region consists of overlapping resonances from glycerophosphocholine (GPC) and glycerophosphoethanolamine (GPE), which are intermediate metabolites of cell membrane degradation [5]. Consequently, PDE is often considered a marker of tissue integrity. Main contributors to the broad peak of membrane phospholipids are considered phosphatidylcholine (PtdCh) and phosphoenolpyruvate (PEP) [6, 7, 8]. PtdCh is the major component of biological membranes, and PEP is a key intermediate in glycolysis and gluconeogenesis. ATP is the central molecule in cellular energy metabolism, and Pi represents one of ATP breakdown products, frequently associated with tissue ischemia. PCr functions as a rapidly accessible energy buffer that regenerates ATP during periods of increased energy demand.

^31^P‐MRS has been used for over three decades to assess metabolite levels in the human kidney [5, 9, 10, 11, 12, 13, 14, 15, 16, 17, 18]. However, the relatively low sensitivity of ^31^P nuclei compared to ^1^H (gyromagnetic ratio 17.2 vs. 42.6 MHz/T), together with the low concentration of ^31^P metabolites, results in intrinsically weak signals. As a result, renal ^31^P‐MRS typically requires signal averaging over a relatively large kidney volume. The primary clinical applications include noninvasive assessment of renal viability prior to transplantation [9, 12, 14] and investigation of causes of graft dysfunction [3, 11, 15]. ^31^P‐MRS of transplanted kidneys is generally more feasible than measurements in native kidney, as the signal‐to‐noise ratio (SNR) per unit time is higher. This improvement is mainly due to the more superficial location of transplanted allografts relative to the surface coil, compared with orthotopic kidneys. Historically, ^31^P‐MRS at 1.5T has been widely used to evaluate post‐transplant renal dysfunction and to monitor therapeutic responses. Conditions such as acute rejection episodes (ARE), chronic (late) kidney graft dysfunction (LGD), acute tubular necrosis (ATN), and cyclosporine nephrotoxicity have been investigated using this approach [3, 5, 10, 11, 13].

In contrast, ^31^P‐MRS of the kidney in healthy volunteers remains technically challenging due to the relatively large distance (60–80 mm) between the kidney and the transmit‐receive ^31^P surface coil. Despite these challenges, phosphorus spectra of the normal kidneys are of considerable importance, as they provide essential reference data for interpreting the spectra in studies of kidney physiology and disease, as well as for the evaluation of human allograft viability and function before and after transplantation. To date, reports of ^31^P spectra from healthy human kidneys in situ are scarce and were published more than three decades ago [10, 16, 17, 18]. These early studies were performed on 1.5 and 2T systems using image‐selected in vivo spectroscopy (ISIS) [19] and three‐dimensional chemical shift imaging (3D‐CSI).

The aim of the present study was to investigate whether ^31^P spectra of orthotopic kidneys can be acquired with improved spectral quality within a clinically acceptable measurement time using a modern 3T MR scanner.

## 2 Methods

### 2.1 Subjects

Six individuals (four female and two male) accepted for kidney donation were included in the study. Median age was 62 years (range, 50–67) and median body mass index (BMI) was 26 kg/m^2^ (range, 21–29). All participants underwent evaluation according to the national protocol for living kidney donor assessment and were considered healthy. Clinical and biochemical characteristics of the study participants prior to donation are presented in Table 1. The study was approved by the Swedish ethical authority committee of Stockholm (dnr 2021‐04897, approved 21‐10‐06). Written consent was obtained from all participants, and all procedures were performed in accordance with the Declaration of Helsinki.

**TABLE 1:** Donor clinical and biochemical characteristics at baseline pre‐donation.

Variables	Donor 1	Donor 2	Donor 3	Donor 4	Donor 5	Donor 6	Summary median (IQR)
Sex	Female	Female	Male	Female	Female	Male	4F/2M
Age (years)	50	67	53	60	64	67	62 (53–67)
BMI (kg/m^2^)	27	21	26	29	26	23	26 (23–27)
SBP (mmHg)	120	120	140	129	126	120	123 (120–129)
DBP (mmHg)	82	74	80	77	76	60	76 (74–80)
CRP (mg/L)	1.1	7.9	0.3	1.2	0.6	0.6	0.9 (0.6–1.2)
Fasting glucose (mmol/L)	5.8	5.8	6.2	5.5	6.2	5.6	5.8 (5.6–6.2)
eGFR (creatinine, mL/min/1.73 m^2^)	77	66	71	57	79	80	74 (66–79)
mGFR (iohexol, mL/min)	95	105	88	78	80	84	86 (80–95)
UACR^a^ (mg/mmol)	0.3	0.5	0.3	0.3	1.0	0.3	0.3 (0.3–0.5)
Regular medication	Estrogen	Prednisolone and PPI	None	None	None	None	—

Abbreviations: CRP, C‐reactive protein; DBP, diastolic blood pressure; eGFR, estimated glomerular filtration rate; Median (IQR), median interquartile range; mGFR, measured glomerular filtration rate; SBP, systolic blood pressure; UACR, urinary albumin‐creatinine ratio.

^a^ Mean value of three UACR measurements.

### 2.2 Data Acquisition

All examinations were performed on a 3T MR system (Achieva dStream, Philips Healthcare, Best, the Netherlands) using a circular transmit‐receive ^31^P surface loop coil (diameter 140 mm), manually tuned, with a maximum *B*_1_ amplitude (*B*_1max_) of 60 μT at the coil center. Volunteers were positioned supine, head‐first. Balanced turbo field echo (BTFE) images [20, 21] were acquired in the transversal, sagittal, and coronal planes and used for spectroscopic voxel placement in the right kidney. Single‐voxel spectra were obtained during free breathing using a 3D‐ISIS localization sequence (TR 5000 ms, 512 averages, spectral bandwidth 3000 Hz, 2048 complex points), resulting in a net acquisition time of 43 min. The voxel size was chosen to be as large as feasible within the boundaries of the kidney. Second‐order pencil beam shimming was applied to improve static magnetic field homogeneity within the voxel [22]. The basic 3D‐ISIS sequence was augmented with broad‐band proton (^1^H) decoupling and nuclear Overhauser effect (NOE) enhancement. The whole‐body coil was used for imaging, decoupling, and NOE enhancement. For spatial localization (slice selections), HS adiabatic full‐passage inversion pulses (duration, 4.22 ms; bandwidth of 2.95 kHz) were applied. RF excitation was performed using a hyperbolic‐secant (HS) adiabatic pulse (duration, 5.42 ms; bandwidth of 1.15 kHz). NOE enhancement was achieved using narrow‐band irradiation (mixing time, 4100 ms; frequency offset, −100 Hz relative to the water resonance; *B*_1max_, 0.5 μT). Proton decoupling was performed by WALTZ‐4 phase‐cycled broadband pulses with a frequency offset of −100 Hz.

HS adiabatic pulses exhibit a decrease in effective amplitude with increasing distance from the surface coil, resulting in a reduced effective excitation bandwidth. Therefore, the transmitter excitation profile was measured as a function of distance between the voxel center and the surface coil. This was performed using a spherical phantom (diameter, 100 mm) filled with 50‐mM monopotassium phosphate (KH_2_PO_4_) aqueous solution. A 30 × 50 × 50 mm^3^ voxel was positioned parallel to the coil, and the Pi signal intensity was recorded as a function of transmitter carrier frequency.

Although phosphocreatine (PCr) is not present in renal tissue, a PCr spectral line was observed in the acquired spectra. This signal originates from adjacent skeletal muscles and is attributed to partial volume effects and respiratory motion of the kidney. Intracellular pH was estimated from the chemical shift difference between Pi and PCr spectral lines [3] according to the widely used Henderson–Hasselbalch formula [23]. The contribution of muscle‐originated ^31^P signals to the kidney spectrum was estimated by measuring a spectrum from calf muscle using identical acquisition parameters and 128 averages.

### 2.3 Spectrum Processing

^31^P spectra were analyzed using the time domain Advanced Method for Accurate, Robust and Efficient Spectral fitting (AMARES) algorithm implemented in the jMRUI software package [24]. Free induction decays (FIDs) were fitted without prior noise suppression. For visualization purposes only, spectra were apodized using a Lorentzian exponential filter with 8‐Hz line broadening. Because renal tissue does not contain phosphocreatine (PCr), the chemical shift axis was referenced to the phosphoethanolamine (PE) singlet at 6.77 ppm, selected for its sharpness, high signal amplitude, and fitting reliability [25]. Peak positions for PE, Pi, GPC, membrane phospholipids (MP), PCr, and γ‐ and α‐ATP were estimated using AMARES. Soft constraints <1.95; 2.3> and <−0.05; 0.05> ppm were applied for membrane phospholipids (MP) and PCr positions, respectively. The splitting of the γ‐ and α‐ATP doublets was fixed to 16 Hz [26], with amplitude ratio of 1:1. The chemical shift of PC relative to the PE line was set to −27.92 Hz, and the frequency difference between GPC and GPE was fixed at 28.44 Hz [25]. Linewidths of PC, GPC, and γ‐ATP were estimated by AMARES. The linewidth of Pi was constrained to 10–30 Hz, and that of MP to 25–35 Hz. Additional constraints were imposed such that the linewidth of PC was equal to that of PE, GPE to GPC, and γ‐ATP to PCr. The linewidths of α‐ATP lines were fixed relative to γ‐ATP, with a scaling factor of 0.8. The relative phase of all spectral lines was fixed to zero. Zero‐ and first‐order phase corrections were estimated by AMARES, with first‐order phase restricted to ±0.5 ms in the time domain. Individual spectra were frequency‐ and phase‐corrected using the described prior knowledge and added together [25]. Only the most reliable spectral lines: PME (PE + PC), Pi, PDE (GPE + GPC), and γ‐ATP were used for computing the spectral intensity ratios. Spectral intensity fractions (%) were computed as the fraction of summed PME, Pi, PDE, MP, and γ‐ATP. The PCr peak was excluded from quantification, as it originates from surrounding skeletal muscle. The α‐ATP, NAD, and β‐ATP intensities were also excluded from quantification due to a marked reduction in signal intensity caused by the transmitter excitation profile and increased chemical shift displacement errors (CSDEs). Quantitative results were corrected for the transmitter excitation profile [23].

### 2.4 Statistics

The reported values are expressed as the means ± 1 SD or as medians and interquartile range (IQR).

## 3 Results

**FIGURE 1:**
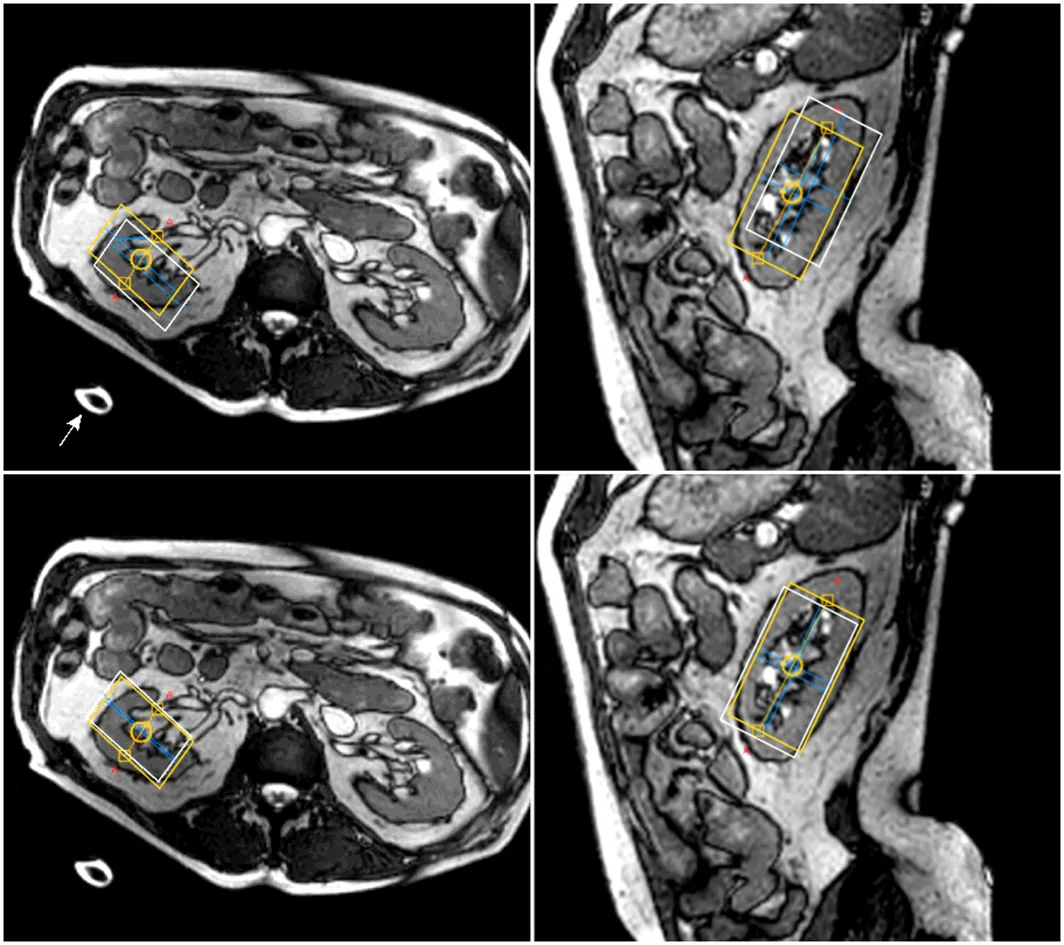
Example of voxel positioning (35 x 60 x 85 mm^3^) shown in axial and sagittal planes. The transmitter carrier frequency corresponds to the yellow voxel. White rectangles indicate voxel positions associated with the PE (upper images) and γ‐ATP (bottom) resonances. The centre of the surface coil is indicated by a small water‐containing bottle (arrow).

**FIGURE 2:**
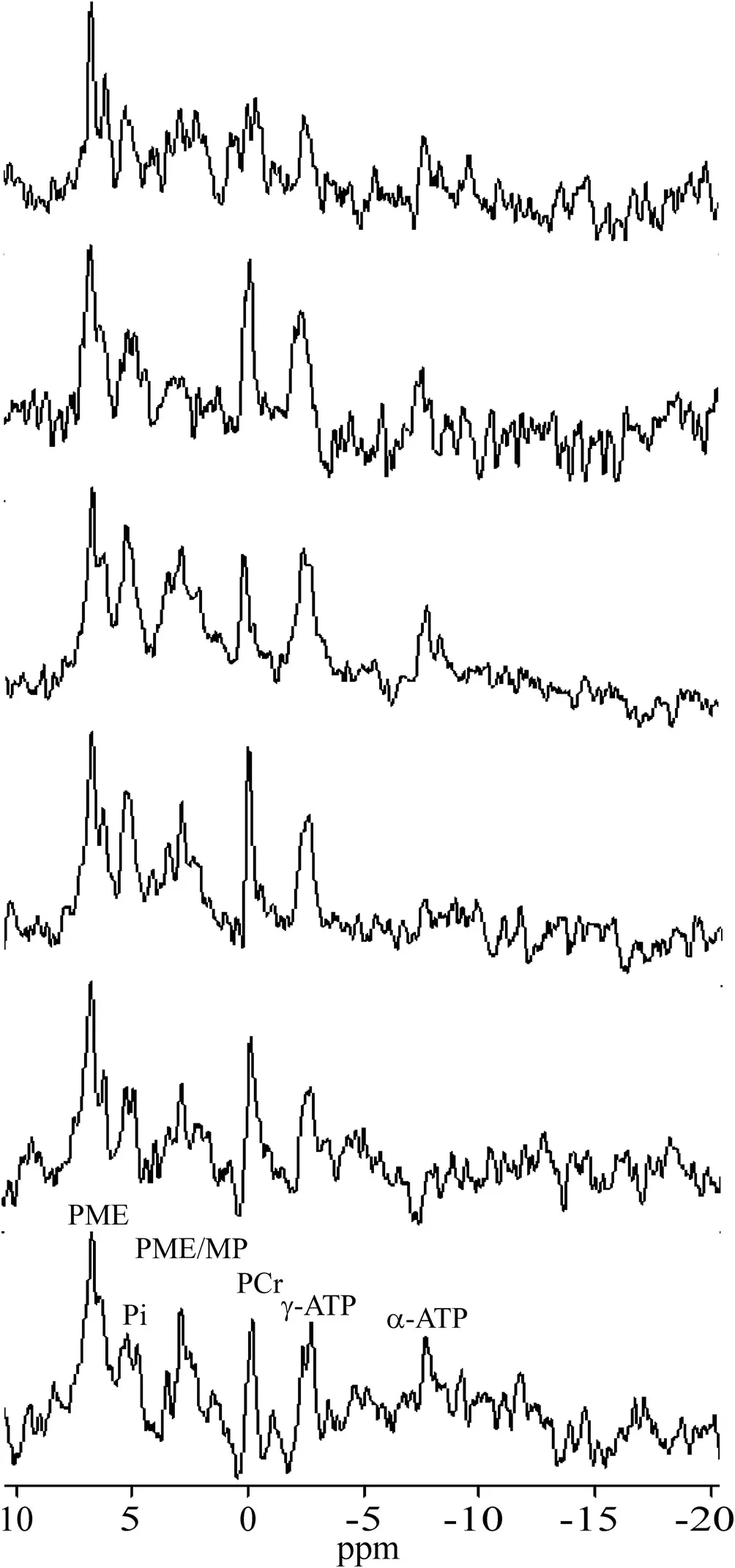
Individual kidney spectra acquired of all donors 1 day before donation.

**FIGURE 3:**
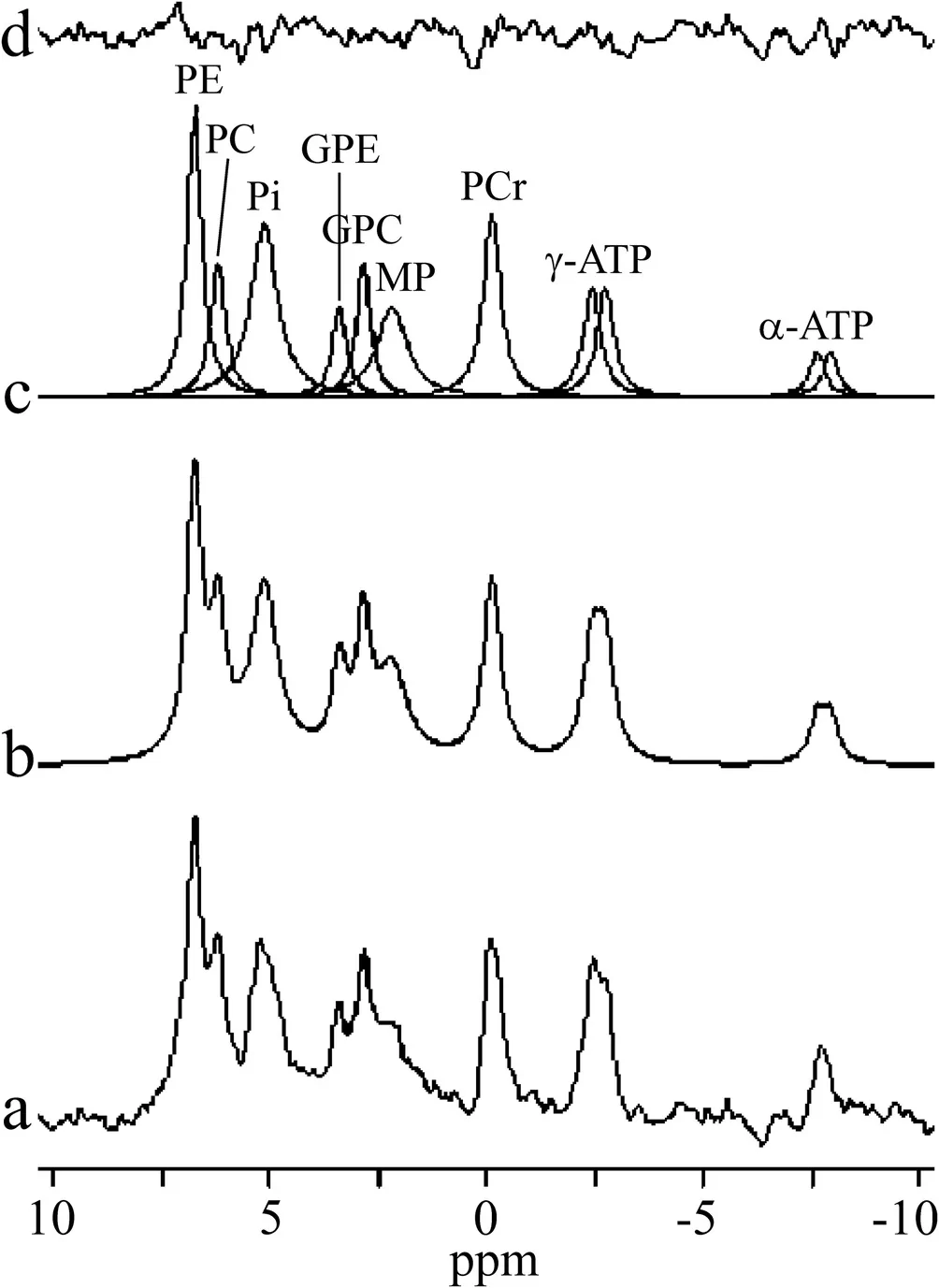
^31^P spectrum of the normal human kidney in situ. (a) The added spectrum of the six healthy volunteers (Figure 2), (b) fitted spectrum, (c) individual components, and (d) residue.

**FIGURE 4:**
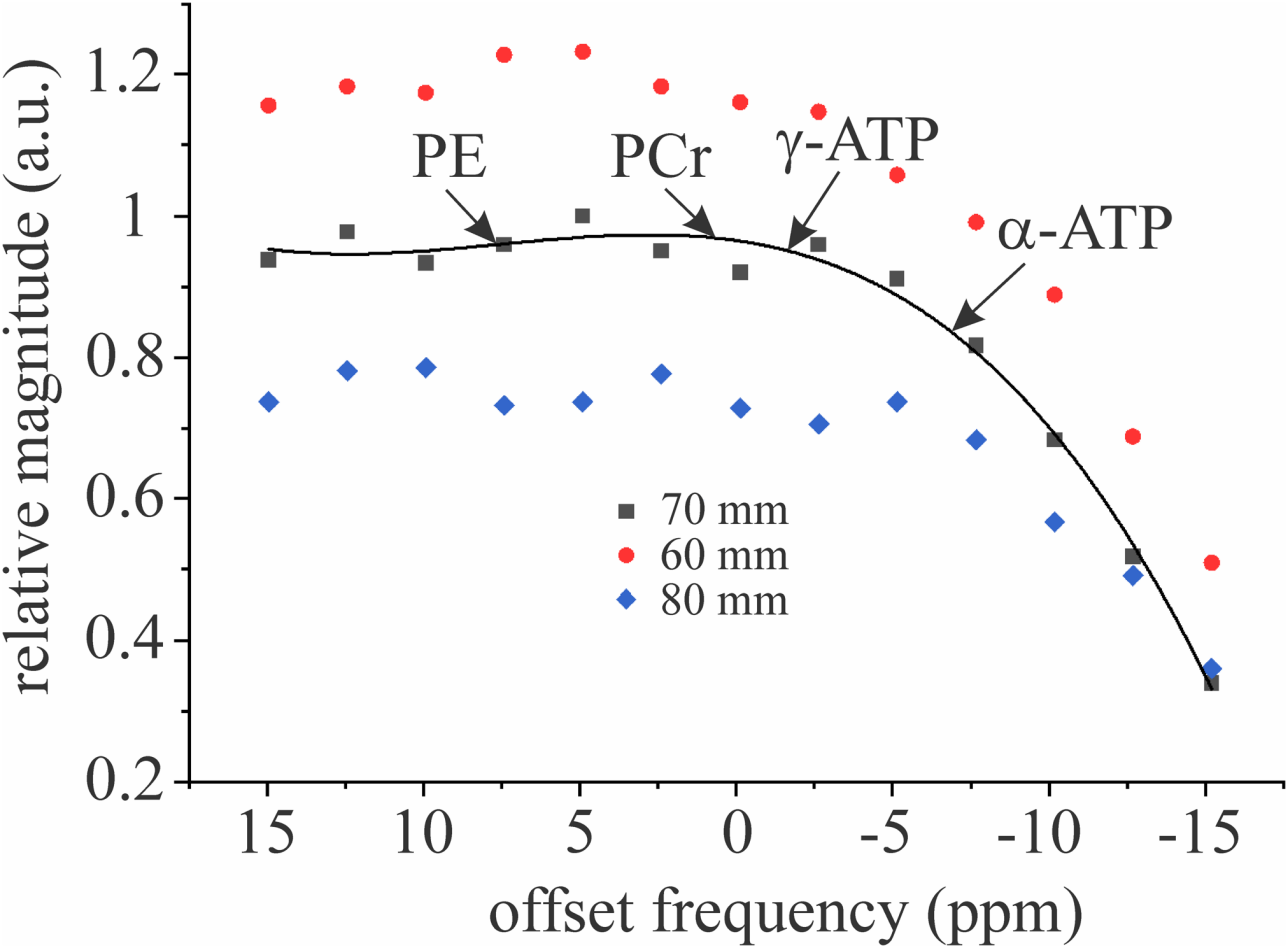
The transmitter excitation profile for 60‐, 70‐, and 80‐mm distance between the center of the voxel and the surface coil.

Clinical data of the study participants are presented in Table 1. Representative voxel positions corresponding to transmitter carrier frequency, PE, and γ‐ATP spectral lines are shown in Figure 1. The carrier frequency was calculated from the water resonance frequency using the Larmor equation and the gyromagnetic ratios of ^1^H and ^31^P [27]. The carrier frequency voxel is located relatively close to the PCr voxel because the distance between PCr resonance and carrier frequency is only ~0.86 ppm. The typical voxel size was 35 × 55 × 90 mm^3^ in the anterior–posterior, left–right, and foot–head directions, respectively. The average voxel volume was 174 ± 20 cm^3^ (range: 149–204). For typical voxel size, the PE voxel was displaced relative to the carrier frequency voxel by approximately 4 mm posteriorly, 7.5 mm to the left, and 9 mm cranially (Figure 1). The γ‐ATP voxel was shifted by approximately 1.8 mm anteriorly, 3 mm to the right, and 3.5 mm caudally. The displacement of the α‐ATP voxel further increased to 4.8 mm anteriorly, 9.7 mm to the right, and 10.8 mm caudally. The *B*_0_ shimming resulted in a water linewidth of 28.4 ± 2.3 Hz (range: 26–31). The mean distance between the voxel center and the surface coil was 75 ± 4.5 mm (range: 69–80). Figure 2 shows individual kidney spectra acquired of all donors 1 day before donation. The summed spectrum and corresponding fitting results are shown in Figure 3. Cramér–Rao lower bounds (CRLBs) of the spectral line fits were 4%, 7%, 11%, 15%, and 5% for PE, Pi, GPC, MP, and γ‐ATP lines, respectively. The transmitter excitation profile for 60‐, 70‐, and 80‐mm distance between the center of the voxel and the surface coil is shown in Figure 4. These measurements demonstrate an approximately 20% reduction in coil sensitivity between distances 60–70 and 70–80 mm. The spectral intensity ratios and the intensity fractions (%) corrected for the excitation profile [23] are presented in Tables 2 and 3, respectively. The pH value of 7.36 was computed from the summed spectrum (Figure 3). The spectrum of the calf muscles (not shown) was used to estimate contamination of the renal spectra by muscle intensities. The spectral amplitude ratios of the muscle metabolites to PCr were 0.047 (Pi/PCr), 0.067 (GPC/PCr), and 0.054 (γ‐ATP/PCr). These ratios indicate that the infiltration of muscle signals to the kidney spectrum was within the noise level, considering the low PCr peak in the spectra (Figure 2).

**TABLE 2:** Spectral intensity ratios.

PME/Pi	PDE/Pi	γ‐ATP/Pi	PME/γ‐ATP	PDE/γ‐ATP	PME/PDE
1.33^a^	0.65^a^	0.83^a^	1.62^a^	0.79^a^	2.04^a^
1.41 ± 0.35^b^ 1.03–1.95	0.66 ± 0.17^b^ 0.46–0.87	0.78 ± 0.22^b^ 0.54–1.12	1.91 ± 0.59^b^ 1.09–2.66	0.92 ± 0.39^b^ 0.42–1.17	2.39 ± 1.17^b^ 1.19–4.27

^a^ Value acquired from the summed spectrum of all volunteers.

^b^ Mean ± 1 SD and range computed from the individual spectra of the volunteers.

**TABLE 3:** Spectral intensity fractions (%) expressed as the fraction of summed PME, Pi, PDE, MP, and γ‐ATP.

PME	Pi	PDE	PDE + MP	MP	γ‐ATP
30.3^a^	22.7^a^	14.9^a^	28.2^a^	13.3^a^	18.8^a^
31.9 ± 6.3^b^	22.8 ± 1.8^b^	15.1 ± 4.1^b^	27.5 ± 7.7^b^	12.4 ± 5.2^b^	17.7 ± 5^b^
23.4–40	20.5–26	9.4–19.7	16.6–38.8	5.7–20.5	12.1–25.7

^a^ Value acquired from the summed spectrum of all volunteers.

^b^ Mean ± 1 SD and range computed from the individual spectra of the volunteers.

## 4 Discussion

The considerable depth of the orthotopic kidneys in the human body (69–80 mm), combined with respiratory motion, provides a major challenge for ^31^P‐MRS by degrading both SNR and spectral resolution. Early studies performed at 1.5 and 2T using single‐voxel ISIS [10, 16, 17] and 3D‐CSI [18] reported spectra with limited spectral resolution and poor SNR. However, significant technological advances in MR hardware and software over the past three decades have improved the feasibility of in vivo phosphorus spectroscopy. To the best of our knowledge, the present work provides the first demonstration of ^31^P spectra of healthy orthotopic human kidneys acquired at 3T. Furthermore, this is the first report in which kidney 3T spectra were enhanced through the combined use of proton decoupling and NOE enhancement.

Except for the study by Vallée et al. [18], which employed proton decoupling, previous ^31^P‐MRS studies of orthotopic [10, 16, 17] or transplanted human kidneys [5, 11, 13, 28] were performed without proton decoupling. A common limitation of such spectra is reduced spectral resolution due to spin–spin coupling between ^31^P and ^1^H atoms. This phenomenon results in subtle splitting of ^31^P resonances, which manifests as line broadening. Proton decoupling eliminates ^31^P‐^1^H coupling by applying radiofrequency (RF) irradiation at the ^1^H Larmor frequency during acquisition, resulting in narrower singlets with increased intensity. This improves both spectral resolution and SNR, facilitating more reliable identification of metabolites such as PE, PC, GPE, and GPC. Vallée et al. [18] demonstrated that proton decoupling increased PME, Pi, PDE, and ATP amplitudes by more than 30%. Additional enhancement of spectral line amplitudes can be achieved by NOE. This method utilizes dipolar interactions between neighboring ^31^P and ^1^H spins by applying RF irradiation to ^1^H spins. However, unlike proton decoupling, where RF irradiation is applied during ^31^P signal acquisition, NOE irradiation is applied prior to ^31^P signal acquisition. Saturation of the ^1^H spin system leads via ^31^P‐^1^H cross‐relaxation to an increased population difference of ^31^P spins, thereby enhancing signal intensity. At 3T, NOE enhancement can further increase ^31^P spectral intensities in the range of 15%–50% [29].

In the present work, the combined use of proton decoupling and NOE enhancement resulted in substantial improvements in both SNR and spectral resolution. Our spectra differ markedly from previously published spectra of normal [10, 16, 17] and well‐functioning transplanted kidneys [5, 11, 13, 28] acquired without proton decoupling and NOE enhancement. In those studies, PME signals were typically less intense than Pi and PDE. However, our spectra are in good agreement with the proton‐decoupled spectra of Vallée et al. [18]. The NOE enhancement used in our experiments, combined with proton decoupling, further increased spectral line amplitudes. In particular, the PE resonance dominates, and individual contributions from PC, GPE, and MP are clearly resolved (Figure 3). In contrast, α‐ and especially β‐ATP resonances exhibit reduced intensity. This is attributed to increased CSDEs and the decline of the RF excitation profile in this spectral region (Figure 4). Consequently, our quantitative analysis was restricted to the spectral interval from PME to γ‐ATP, where the excitation profile is relatively uniform. Within this region, partial volume effects due to chemical shift displacement of PE and γ‐ATP voxels were minimized through careful voxel placement (Figure 1). It should also be noted that γ‐ATP serves as a reliable representative of ATP content, as the γ‐, α‐, and β‐ATP resonances are theoretically of equal intensity. Potential contamination of the renal spectra from surrounding muscles was carefully evaluated. Contributions from skeletal muscle, assessed via calf muscle measurements, were found to be negligible due to the relatively small PCr signal in the renal spectra. Other potential sources include urine Pi and blood (erythrocytes) compound 2,3‐diphosphoglycerate (2,3‐DPG) [2]. Urine Pi may overlap PDE signals (~3.25 ppm) due to the low pH (5.2 ± 0.2) of urine [30, 31]; however, a previous study by Grist et al. [32] has demonstrated minimal contribution in the absence of urinary obstruction. Similarly, the 2,3‐DPG doublet (6.24 and 5.24 ppm) overlaps with PME and Pi resonances [27, 33]. The strong PME and Pi intensities obscure these spectral lines, making them difficult to resolve. Previous studies demonstrated that the contribution of 2,3‐DPG to the kidney spectrum is negligible, as its concentration in the blood is low and the vascular volume in a well‐functioning kidney is relatively small (ca. 20%) [2, 10, 18, 34].

The intracellular pH value was estimated for completeness because of the well‐resolved Pi and PCr spectral lines in the summed spectrum (Figure 3). This estimate should be interpreted with caution as the PCr signal originates from tissues surrounding the kidney. Nevertheless, the estimated pH value of 7.36 agrees well with the reported value of 7.29 ± 0.23 for well‐functioning transplanted kidneys [3].

The main limitation of this work, as well as previous studies, is the relatively large voxel size required to achieve adequate SNR. As a result, the acquired spectra represent a composite signal from multiple renal compartments, including cortex, medulla, and pelvis. Since metabolite distribution differs across these regions (PE dominates in cortex, GPC in medulla, and GPE together with Pi can be found in both compartments) [2], careful interpretation is required. Furthermore, the quantitative results are specific to the acquisition protocol and scanner configuration used, as NOE enhancement depends on both methodology and vendor‐specific implementation. Finally, the 3D‐ISIS localization technique is limited by relatively large CSDEs due to the limited bandwidth of excitation pulses and by dependence of the RF excitation profile on distance between surface coil and the voxel.

## 5 Conclusions

The present work demonstrates, to the best of our knowledge, the first acquisition of in vivo ^31^P spectra of the healthy human orthotopic kidney at 3T. The use of a 3D‐ISIS localization sequence in combination with proton decoupling and NOE enhancement enabled acquisition of spectra with improved quality within a clinically acceptable measurement time. The combined effects of CSDEs and the nonuniform transmitter excitation profile limited quantitative analysis to the spectral region from PME to γ‐ATP. Reference ^31^P spectra of healthy orthotopic kidney under these conditions may provide a valuable baseline for assessing alterations in renal metabolism associated with disease and transplantation.

## Author Contributions

All authors contributed to conception and design of the study. J.W. and M.J. provided the MRS sequence protocol. E.C., A.S., P.L., and M.K.S. collected clinical data and managed kidney donor participation. J.W. performed ^31^P‐MRS acquisitions and spectrum processing with support from M.J. E.C. performed the statistical analyses. All authors contributed to data interpretation. J.W. drafted the manuscript, with critical intellectual input from A.S., P.L., E.C., M.J., and M.K.S. All authors reviewed and approved the final manuscript. M.K.S., A.S., and P.L. provided overall supervision.

## Funding

This work was supported by the ALF Funding from Uppsala University and establishment funding (M.K.S.), the CUWX Foundation, the Swedish Kidney Foundation, and the Swedish Society of Nephrology.

## Conflicts of Interest

The authors declare no conflicts of interest.

## Data Availability

The data that support the findings of this study are available on request from the corresponding author.
